# A case of otitis media complicated by intracranial infection with *Actinomyces turicensis*

**DOI:** 10.1099/jmmcr.0.004408

**Published:** 2014-12-01

**Authors:** Sarah Miller, Tony Walls, Neil Atkinson, Sona Zaleta

**Affiliations:** ^1^​ Timaru Hospital, South Canterbury, New Zealand 7190; ^2^​ Christchurch Hospital, Riccarton Avenue, Christchurch, New Zealand

**Keywords:** Keywords: *Actinomyces turicensis*, antibiotics, cerebellar abscess, neurosurgery, otitis media.

## Abstract

**Introduction:**

Actinomycosis is a granulomatous suppurative infection caused by filamentous Gram-positive anaerobic bacteria from the family *Actinomycetaceae*. To our knowledge, this is the first reported case of otogenic brain abscess associated with *Actinomyces turicensis*.

**Case presentation:**

We report the case of an immunocompetent 5-year-old boy with recurrent otitis media who re-presented to the emergency department with a 3-week history of otorrhoea, progressive anorexia, vomiting and lethargy. He was admitted with a working diagnosis of otitis media and dehydration, and was treated with intravenous fluids and oral co-trimoxazole. He subsequently developed abnormal posturing with a reduced Glasgow coma score and seizures. Urgent computed tomography revealed a cerebellar abscess with obstructive hydrocephalus for which he underwent urgent neurosurgical intervention. Tissue and aspirate cultures revealed a polymicrobial infection with *A. turicensis*. The patient has since undergone long-term antibiotic treatment and has made a good recovery.

**Conclusion:**

This case demonstrates the successful use of long-term antibiotic therapy and neurosurgical intervention to treat otogenic brain abscess associated with *A. turicensis* infection. To the best of our knowledge, this is the first such documented case. Our report also provides a timely reminder that, despite a reduced incidence in the developed world, intracranial complications of otitis media continue to occur and a high index of suspicion is required.

## Introduction

Actinomycosis is a slowly progressive granulomatous disease caused by filamentous Gram-positive anaerobic bacteria from the family *Actinomycetaceae*. Commensals of the oropharynx, gastrointestinal tract and urogenital tract, *Actinomyces* spp. can become pathogenic. *Actinomyces israelii* is considered the most common human pathogen in this family and the orocervicofacial, thoracic and abdominopelvic sites are those most commonly affected (Wong *et al.*, 2011). Intracranial infection with *Actinomyces* spp. is rare, and there are a limited number of cases reported in the literature. Diagnosis is difficult, and there remains no consensus about treatment of cerebral actinomycosis. This report discusses the presentation, diagnosis and management of a case of otitis media complicated by intracranial infection with the species *Actinomyces turicensis*, which, to the best of our knowledge, has not been documented previously.

## Case report

A 5-year-old boy with a background of recurrent otitis media re-presented to the emergency department with a 3-week history of ongoing otorrhoea with progressive anorexia, vomiting and lethargy. He had been seen multiple times in the proceeding 3 weeks by both his GP and the emergency department and had initially been given a course of oral amoxicillin. Swabs from his otorrhoea had grown *Proteus mirabilis* and meticillin-resistant *Staphylococcus aureus* (MRSA), and he had also therefore been given a course of oral co-trimoxazole and regular gentamicin/dexamethasone ear drops. There was no history to suggest that he was immunocompromised.

On examination, observations were stable and he was apyrexial with a temperature of 36.7 °C, although his parents reported fevers at home. He was alert and rousable but appeared in significant pain and was 5 % dehydrated. His right ear contained thick pale green exudate and, when microsuctioned, revealed an inflamed auditory canal. The tympanic membrane was intact but with fluid behind it. Overall appearances were felt to be consistent with otitis media. There was no cervical lymphadenopathy, and examination of the chest and abdomen was normal. Initial neurological examination revealed no focal neurological signs and no evidence of meningism. Pupils were equal and reactive.

**Fig. 1.**
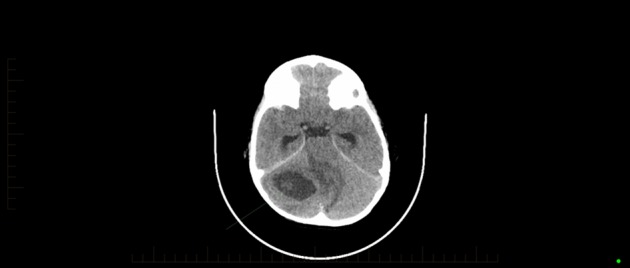
Initial CT imaging demonstrating a cystic mass in the right cerebellar hemisphere with surrounding oedema and mass effect.

**Fig. 2.**
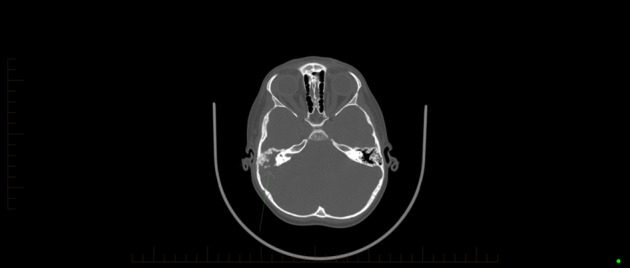
Initial CT imaging demonstrating ossified right middle ear and mastoid air cells, bony irregularity and cortical destruction consistent with osteomyelitis.

The patient was diagnosed with ongoing otitis media and dehydration. Intravenous (i.v.) fluids were commenced and oral co-trimoxazole continued. Overnight, nursing staff noted an episode of decerebrate posturing and decreased responsiveness. Upon assessment by the on-call doctor, his Glascow coma score was 13 (E3, M6 and V4) and the posturing had ceased. Pupils were equal and reactive but with a bilateral fixed downward left lateral gaze. The patient underwent an urgent computed tomography (CT) scan of the head, the findings of which were consistent with a right cerebellar abscess secondary to complicated severe right middle ear infection (Figs 1 and 2). There was associated obstructive hydrocephalus.

## Investigations

Initial bloods at presentation showed a neutrophilic lymphocytosis (white cell count 15.1×10^9^ l^−1^, neutrophils 13.4×10^9^ l^−1^) with a mildly elevated C-reactive protein (23 mg l^−1^). Renal function was normal (urea 5.6 mmol l^−1^, creatinine 29 µmol l^−1^). Blood cultures did not yield significant growth.

The urgent CT of the head revealed a cystic mass in the right cerebellar hemisphere, measuring 28×40×17 mm, with surrounding oedema and significant mass effect distorting the brainstem to the left and resulting in obstructive hydrocephalus. The right middle ear and mastoid air cells were completely ossified with adjacent bony irregularity and cortical destruction consistent with osteomyelitis complicating a severe middle ear infection (Figs 1 and 2).

Tissue from the right mastoid obtained from later operative intervention grew *A. turicensis*, *Proteus mirabilis* and *Peptoniphilus harei* with cerebellar aspirates positive for *Bacteroides thetaiotaomicron* and *Anaerococcus hydrogenalis* in addition. No MRSA was isolated from deep-tissue samples.

A presumptive identification of an *Actinomyces* sp. was made on the basis of typical Gram-stained findings of Gram-positive branching filamentous rods. The classical sulfur granules were not seen in this case, but further presumptive evidence for actinomycoses was provided by matrix-assisted laser desorption/ionization mass spectrometry of the cultured organism, suggesting *A. turicensis*. This was confirmed by sequencing of the 16S rRNA gene by PCR.

## Treatment

Immediately after obtaining the first series of non-contrast CT images, the patient had a generalized seizure that responded promptly to i.v. midazolam. Following this, he was maintaining his own airway but responding only to painful stimuli. He was transferred to the intensive care unit where he was intubated and ventilated. Sedation was maintained with propofol and midazolam infusions. He was loaded with i.v. phenytoin and a mannitol infusion was commenced. I.v. vancomycin and cefotaxime were given in addition to i.v. dexamethasone. Once stabilized, the patient was transferred to a tertiary centre for neurosurgical intervention.

At the tertiary centre, the patient underwent drainage of the cerebellar abscess with external ventricular drain (EVD) insertion and right mastoidectomy. It was felt that otitis media had caused cholesteatoma formation and subsequent mastoiditis, which in turn led to secondary intracranial pathology. A right lateral gaze palsy was noted post-operatively, which was likely to be iatrogenic. Antibiotics were changed to i.v. cefotaxime and metronidazole in view of the mastoid tissue and cerebellar aspirate culture results. Following removal of the EVD, the patient developed signs of raised intracranial pressure and subsequently underwent redrainage of the abscess with insertion of a second EVD, which was removed 12 days later. The patient was discharged home 5 weeks after presentation with a continuous ambulatory 24-h benzylpenicillin infusion, oral ciprofloxacin and metronidazole.

## Outcome and follow-up

Repeat magnetic resonance imaging a month following discharge showed a significant improvement in the appearance of the posterior fossa with resolution of the mass effect but mild residual signal abnormality and enhancement in the region of the abscess. Revision right tympanomastoidectomy was performed uneventfully and included the removal of the remainder of the cholesteatoma. Antibiotic cover was switched to oral amoxicillin and ciprofloxacin ear drops after this procedure. It is anticipated that the patient will continue oral amoxicillin for 6 months.

In terms of acquired disability, the patient was noted to have a mildly ataxic gait with a lean to the right when walking. At his most recent follow-up, 3 months post-operative, this had almost completely resolved. His right lateral gaze palsy persists. There has been no gross acquired learning disability or deafness and the patient is now back at school.

## Discussion

Actinomycosis is a granulomatous suppurative infection caused by filamentous Gram-positive anaerobic bacteria from the family *Actinomycetaceae* (Gazzano *et al.*, 2010; Wong *et al.*, 2011). These bacteria are commensals of the human oropharynx, gastrointestinal tract and urogenital tract but can become pathogenic when tissue integrity is breached and direct or haematogenous spread occurs (Akhaddar *et al.*, 2010; Fabbri *et al.*, 2014; Wong *et al.*, 2011). Infection is frequently polymicrobial, and the most commonly affected sites are the cervicofacial region, abdomen and thorax (Wong *et al.*, 2011; Gazzano *et al.*, 2010). Cerebral infection, as in our patient, is rare, and indeed there are a limited number of cases of cerebral actinomycosis and fewer than 30 cases of tympanomastoid actinomycosis infection reported in the English language literature (Fabbri *et al.*, 2014; Gazzano *et al.*, 2010).

Of more than 30 *Actinomyces* species, *A. israelii* is the most common human pathogen (Fabbri *et al.*, 2014; Wong *et al.*, 2011). To the best of our knowledge, this is the first reported case of intracranial abscess associated with the species *A. turicensis*. The case is also remarkable in that actinomycosis infection is most common in the third decade of life and is particularly rare in children (Gazzano *et al.*, 2010; Puzzilli *et al.*, 1998). Olah *et al.* (2004) described a 10-year-old boy with a background of complex congenital heart disease who was diagnosed with cerebral abscess secondary to *A. israelii*. This patient also underwent neurosurgical intervention and was successfully treated with a 4-week course of ceftriaxone. In contrast to this, our patient was immunocompetent with no predisposing factors such as head trauma, dental procedures, diabetes or congenital heart disease.

The diagnosis of actinomycosis infection can be difficult (Akhaddar *et al.*, 2010; Wong *et al.*, 2011). Clinically, it may mimic tuberculosis, nocardiosis and malignancies (Fabbri *et al.*, 2014; Wong *et al.*, 2011). Blood tests may show a non-specific leucocytosis and raised C-reactive protein. CT and magnetic resonance imaging also tend to be non-specific in the early stages of infection; however, later they may demonstrate characteristic sinus tract formation and infiltration of surrounding tissue across tissue planes (Wong *et al.*, 2011). The slow-growing nature of *Actinomyces* spp. means that growth in culture may take up to 3 weeks (Roth & Ram, 2010). Combined with its sensitivity to oxygen, this means that making an actinomycosis culture diagnosis can be challenging (Gazzano *et al.*, 2010; Roth & Ram, 2010). Alternatively, a histological diagnosis may be sought through demonstration of Gram-positive filamentous organisms and sulfur granules (Wong *et al.*, 2011). Faster and more accurate identification of *Actinomyces* spp. is available through newer molecular genetic methods such as PCR and 16S rRNA gene sequencing (Wong *et al.*, 2011).

Optimal treatment for actinomycosis infection remains unclear (Akhaddar *et al.*, 2010; Fabbri *et al.*, 2014; Roth & Ram, 2010). Frequently, an aggressive approach, as seen in our case, involving a combination of surgery and long-term antibiotic treatment is taken (Akhaddar *et al.*, 2010; Gazzano *et al.*, 2010; Roth & Ram, 2010; Wong *et al.*, 2011). Following a review of five cases of intracranial actinomycosis infection, Akhaddar *et al.* (2010) proposed aspiration of all abscesses larger than 2 cm, and craniotomy for abscesses that enlarge over 2 weeks or do not shrink after 4 weeks of antibiotic therapy. With regard to antibiotic treatment, high-dose i.v. penicillin G is traditionally used for 2–6 weeks followed by oral penicillin V for 6–12 months (Wong *et al.*, 2011). *Actinomyces* spp. have, however, been shown to be susceptible to a much wider range of antimicrobials including β-lactams, clarithromycin, erythromycin, doxycycline and clindamycin, although not all of these are thought to provide good central nervous system penetration (Smith *et al.*, 2005). Modern approaches therefore now tend to individualize the type and length of antibiotic treatment based on the site and severity of infection as well as patient response (Wong *et al.*, 2011). Metronidazole is not effective against pathogenic *Actinomyces* spp. (Smith *et al.*, 2005); however, it was used with cefotaxime in our patient due to the polymicrobial nature of his infection. The use of ciprofloxacin was advocated by Akhaddar *et al.* (2010), who reported an excellent response in the four patients in their case series in whom it was used. Finally, Puzzilli *et al.* (1998) commented that treatment of intracranial actinomycosis in juvenile patients should not differ from that in other age groups. This report therefore also adds to the scant literature available that supports their conclusion.

In conclusion, this case demonstrates the successful use of tailored long-term antibiotic therapy, combined with neurosurgical intervention, to treat otogenic brain abscess associated with *A. turicensis* infection, which, to the best of our knowledge, has not been documented previously. The development and use of antibiotics mean that the incidence of intra- and extracranial complications of otitis media in the developed world is now low (Agrawal *et al.*, 2005; Daniero *et al.*, 2012; Smith & Danner, 2006). This case therefore also serves as a timely reminder that, despite this, complications do continue to occur and that a high index of clinical suspicion is required.

## Abbreviations

Abbreviations: CT: computed tomography
EVD: external ventricular drain
i.v.: intravenous
MRSA: meticillin-resistant *Staphylococcus aureus*.

## References

[r1] Agrawal S Husein M MacRae D **(**2005**).** Complications of otitis media: an evolving state. J Otolaryngol 34, S33–S39. 16089238

[r2] Akhaddar A Elouennass M Baallal H Boucetta M **(**2010**).** Focal intracranial infections due to *Actinomyces* species in immunocompetent patients: diagnostic and therapeutic challenges. World Neurosurg 74, 346–350. 10.1016/j.wneu.2010.05.029 21492568

[r3] Daniero J Clary M O’Reilly R **(**2012**).** Complications of otitis media. Infect Disord Drug Targets 12, 267–270. 10.2174/187152612801319294 22338586

[r4] Fabbri G Guardigni V Sarubbo S Cultera R Contini C **(**2014**).** Brain abscess sustained by *Actinomyces meyeri* in an immunocompetent patient. Neurol Neurophysiol 5, 184.

[r5] Gazzano E Chanteret C Duvillard C Folia M Romanet P **(**2010**).** A case of actinomycosis of the middle ear and a review of the literature. Int J Pediatr Otorhinolaryngol Extra 5, 70–73. 10.1016/j.pedex.2009.03.001

[r6] Olah E Berger C Boltshauser E Nadal D **(**2004**).** Cerebral actinomycosis before adolescence. Neuropaediatrics 35, 239–241. 10.1055/s-2004-820895 15328564

[r7] Puzzilli F Salvati M Ruggeri A Raco A Bristot R Bastianello S Lunardi P **(**1998**).** Intracranial actinomycosis in juvenile patients. Case report and review of the literature. Childs Nerv Syst 14, 463–466. 10.1007/s003810050262 9808256

[r8] Roth J Ram Z **(**2010**).** Intracranial infections caused by *Actinomyces* species. World Neurosurg 74, 261–262. 10.1016/j.wneu.2010.06.011 21492552

[r9] Smith A Hall V Thakker B Gemmell C **(**2005**).** Antimicrobial susceptibility testing of *Actinomyces* species with 12 antimicrobial agents. J Antimicrob Chemother 56, 407–409. 10.1093/jac/dki206 15972310

[r10] Smith J Danner C **(**2006**).** Complications of chronic otitis media and cholesteatoma. Otolaryngol Clin North Am 39, 1237–1255. 10.1016/j.otc.2006.09.001 17097444

[r11] Wong V Turmezei T Weston V **(**2011**).** Actinomycosis. Br Med J 343, d6099 10.1136/bmj.d6099 21990282

